# Control of Bone Matrix Properties by Osteocytes

**DOI:** 10.3389/fendo.2020.578477

**Published:** 2021-01-18

**Authors:** Amy Creecy, John G. Damrath, Joseph M. Wallace

**Affiliations:** ^1^Department of Biomedical Engineering, Indiana University-Purdue University at Indianapolis, Indianapolis, IN, United States; ^2^Weldon School of Biomedical Engineering, Purdue University, West Lafayette, IN, United States

**Keywords:** perilacunar remodeling, lacunocanalicular network, extracellular matrix, collagen, mineral, mechanical loading

## Abstract

Osteocytes make up 90–95% of the cellular content of bone and form a rich dendritic network with a vastly greater surface area than either osteoblasts or osteoclasts. Osteocytes are well positioned to play a role in bone homeostasis by interacting directly with the matrix; however, the ability for these cells to modify bone matrix remains incompletely understood. With techniques for examining the nano- and microstructure of bone matrix components including hydroxyapatite and type I collagen becoming more widespread, there is great potential to uncover novel roles for the osteocyte in maintaining bone quality. In this review, we begin with an overview of osteocyte biology and the lacunar–canalicular system. Next, we describe recent findings from *in vitro* models of osteocytes, focusing on the transitions in cellular phenotype as they mature. Finally, we describe historical and current research on matrix alteration by osteocytes *in vivo*, focusing on the exciting potential for osteocytes to directly form, degrade, and modify the mineral and collagen in their surrounding matrix.

## Introduction

Embedded within the mineralized matrix of bone, osteocytes, a cell population of growing importance in bone biology and medicine, find great longevity despite their apparently isolated location. Osteocytes are increasingly recognized as cells that govern the process of bone remodeling by directing bone forming osteoblasts and bone resorbing osteoclasts. While these actions play an important role in determining the location and time-course of bone remodeling, osteocytes themselves are positioned to readily access immense quantities of bone tissue. Making up over 90% of the cellular content of bone, osteocytes form a rich network of dendrites that communicate with roughly 50 neighboring osteocytes, resulting in a total surface area that greatly exceeds that of osteoblasts and osteoclasts combined. Therefore, any stimulus that triggers osteocytes to directly interact with the bone matrix could have a great positive or negative impact on the overall integrity of bone. In this review, we begin with a brief discussion of how osteocytes direct the activities of osteoblasts and osteoclasts. Next, we cover the important role of the lacunar-canalicular network (LCN) in osteocyte communication and remodeling. Finally, we discuss the exciting potential for osteocytes to directly modify the organic and inorganic components of the bone matrix, which may form an important basis for future treatment strategies aimed at improving bone mass and tissue quality.

## Osteocyte-Directed Matrix Modification by Osteoblasts and Osteoclasts

At the end of their period of bone formation, late-stage osteoblasts are directed *via* unknown cues to either undergo apoptosis or terminal differentiation (1, 2). One option is for osteoblasts to differentiate into quiescent bone lining cells, which cover the bone surface and are thought to mediate remodeling in localized bone areas (1). Some osteoblasts further differentiate into osteocytes. Late osteoblasts transition to early osteocytes by forming dendrites *via* upregulation of the gene E11/gp38, or podoplanin (3). Upregulation of MT1-MMP, a metalloproteinase that cleaves collagen, is also required for osteocyte dendrite formation and maintains cell viability throughout differentiation (4, 5). These findings may suggest that osteocyte embedding is an active, proteolytic process, in contrast to initial studies that suggested osteocyte embedding is a process of passive entrapment within the matrix (6, 7). Recently, however, studies utilizing intravital imaging have suggested that there may be multiple mechanisms for osteocyte embedding that involve some combination of the above processes, as well as lacunar reshaping prior to differentiation (8). Once embedded, the osteocyte begins its role in coordinating the actions of osteoblasts and osteoclasts as a part of the rich osteocyte network.

### Osteocyte Communication With Osteoblasts

The activities of osteoblasts and osteoclasts are highly regulated by signals originating from osteocytes, although the mechanisms by which signals reach these cells are poorly understood. Osteoblasts are responsible for new bone formation, which primarily occurs on trabecular and cortical bone surfaces (9). Bone formation is notably induced by the Wnt signaling pathway. The canonical pathway involves Wnt binding to low-density lipoprotein receptor-related protein 5/6 (Lrp5/6) and its co-receptor, Frizzled (10). This binding inhibits the intracellular activity of glycogen synthase kinase 3 (GSK3) and its complex consisting of Axin and adenomatous polyposis coli (APC), which results in hypophosphorylation of the transcription factor *β*-catenin (11). Translocation of intact *β*-catenin to the nucleus results in the expression of genes that enhance osteoblast survival and bone formation activity. Osteocytes are an important regulator of this process *via* the secretion of Sclerostin (Sost). Sost is a potent suppressor of Wnt signaling by binding Lrp5/6, competitively inhibiting Wnt binding (12). This results in uninhibited phosphorylation of *β*-catenin and its subsequent degradation by the proteasome. Sclerostin has also been shown to inhibit bone morphogenic protein (BMP)-related bone formation (13). In humans, mutations in Sost result in sclerosteosis, a condition characterized by increased bone formation resulting in high bone mass and cranial neuropathies due to nerve compression (14). Therefore, osteocytes have the potential to control when and where bone formation occurs and interfering with this process can have dramatic effects on human health.

### Osteocyte Communication With Osteoclasts

Interestingly, osteocytes also mediate the process of bone resorption by osteoclasts. One method of regulation is through osteocyte secretion of receptor activator of nuclear factor kappa B ligand (RANKL) through their dendrites, which binds the RANK receptor on osteoclast precursors and drives their differentiation into mature osteoclasts (15). RANKL expression by osteocytes is essential for trabecular bone remodeling and is secreted by osteocytes in regions of osteocytic apoptosis (16, 17). Additionally, osteocytes secrete osteoprotegerin (OPG), a molecule that competes with RANKL for the RANK receptor (18). This interaction suppresses osteoclast activity and is the basis for the anti-resorptive osteoporosis drug denosumab (19, 20). Frequently, the overall secreted RANKL/OPG ratio is measured in *in vitro* and *in vivo* models, and in humans, to approximate the degree of osteoclastogenesis in the bone (21). Therefore, osteocytes can modify the total content and activity of mature osteoblasts and osteoclasts, demonstrating their important regulatory role in the process of bone remodeling.

Repair of bone microdamage has also been shown to be dependent on the coordinated actions of osteocytes and osteoclasts. Microdamage, or small cracks or breaks in the bone, trigger osteocyte apoptosis and induce intracortical remodeling, a process that is atypical in rodent cortical bone (22). Further, regions of bone remodeling colocalize with regions of osteocyte apoptosis in the context of microdamage or estrogen deficiency (22, 23). *In vitro* studies have demonstrated that apoptotic osteocytes stimulate their neighbors to release RANKL, which acts as a chemotactic signal for osteoclasts to migrate into the regions of apoptosis and remodel the bone (24). Therefore, osteocytes also utilize osteoclasts to repair regions of microdamage through the controlled release of RANKL while preserving undamaged regions of the bone.

The process of remodeling is slow and deliberate, but evidence demonstrating decreased whole body bone mineral content in lactating women suggests that rapid changes in systemic mineral demands must be met by liberating mineral from the bone (25). Furthermore, bone matrix components must rapidly reform to maintain bone strength when mineral demands are lifted. Indeed, weaning triggers osteoclast apoptosis and a decrease in RANKL levels within the bone while osteoblastic activity remains elevated, favoring bone formation (26). However, considering that the resorption and formation processes by osteoclasts and osteoblasts, respectively, primarily occur on bone surfaces, it is logical that osteocytes may utilize their large surface area to release bone mineral during lactation and reconstruct their surrounding matrix after weaning. Therefore, due to their large population, extensive network, and sprawling surface area, researchers have begun investigating the osteocyte as a potential candidate for rapid bone alterations when subjected to stimuli that alter bone formation and resorption (27). The remainder of this review will focus on observations from *in vitro* and *in vivo* studies examining the potential for osteocytes to control the structure and composition of bone by modulating the activity of osteoblasts and osteoclasts and by direct interaction with the extracellular matrix.

## Lacunar–Canalicular Network and Its Role in Transmitting Mechanical Stimuli

Osteocyte cells are embedded in the mineralized matrix in protected lacunae which surround the cell body. The cells are connected to one another by dendritic cell processes which reside in canaliculi. Together, these form the lacunar–canalicular network (LCN). This interconnected network of cells may be relevant to mechanical sensing and is important for signaling and solute transport (28). As extracellular fluid flows through the LCN, the osteocytes release chemicals such as nitric oxide, prostaglandin, and other factors (29). Additionally, the level of mechanical stimuli is related to osteocyte apoptosis which promotes osteoclastogenesis and is a mechanism by which osteocytes regulate bone repair and shape (28). Loading enhances fluid flow and the shape of the LCN may affect how fluid flows through the system, as observed by the use of injected tracers, where there is an increase in labeled osteocytes with loading (30). Ciani et al. saw an increase in the percentage of osteocytes labeled with an injected tracer in loaded tibiae compared to non-loaded tibiae of rats. However, this only occurred in cancellous bone, not cortical bone (30). This increase of fluid flow with loading has also been speculated with numerical methods. Multiple groups have attempted to quantify the forces placed on the LCN using finite element analysis (FEA) and numerical models which indicate that the shape of the network influences the shear stress the cell is exposed to (31–33). The model in Gatti et al. indicated that vascular porosity plays a role as well, with idealized models showing a decrease in fluid velocity with an increase in vascular porosity (33). Using a fluid–structure interaction model to model a single cell, Joukar et al. indicated cells in rounded lacunae experienced less shear stress than elliptical ones under different modes of loading (32). The overall organization and shape of the LCN affect the ability of the osteocyte to sense stimuli, communicate with other cells, and effectively modulate bone quality.

The LCN can be imaged multiple ways, in two and three dimensions to provide quantitative measures of the LCN and osteocyte shape and organization. Two dimensional methods include scanning electron microscopy (11) with silver staining or quantitative backscatter imaging. Three dimensional methods allow for data analysis on connections between the osteocytes. These include high resolution micro-computed tomography, second harmonic generation, and confocal microscopy when combined with staining (34). In addition to the LCN shape, the osteocyte cell can be imaged using a combination of staining and confocal microscopy. Recent research has utilized green fluorescence protein (GFP)-labeled osteocytes and other dyes to image cellular aspects of the osteocyte such as the cytoskeleton along with the dendritic connections in relation to the surrounding collagen (35). This approach has yielded some evidence that vesicles may be released by the osteocyte as it embeds itself within the LCN and that collagen may be produced by the osteocyte. It is important to note that quantifying the number of lacunae is not the same as quantifying occupied lacunae (36). After an osteocyte dies, the lacunae will remain empty until it is gradually filled with mineralized debris.

Quantitative analysis of the LCN structure must be done to determine if alterations to the shape and organization of the network are occurring. Lacunar area or volume, lacunar density, canalicular length, and canalicular density are some of the measurements that can be made to quantify changes. The LCN can also be quantified in a manner similar to quantification of the connectivity of the trabecular bone network. This is essentially a measure of how many connections would have to be broken to separate the network into two (37). Additionally, the LCN can be analyzed in terms of connectomics. In this analysis, the LCN is considered as a system of nodes linked together by edges. Nodes can be either lacunae or places where at least three canaliculi connect. This analysis could be useful to determine how the organization of the LCN affects the osteocyte’s ability to communicate with other cells and respond to loading. Nodal centers with higher numbers of connections may indicate fewer and more utilized routes of communication. Connectomics analysis has been reviewed in depth elsewhere (38). Less work has been done using connectomics analysis, but there have been some studies that have utilized this technique. Mabilleau et al. have indicated that high fat diet caused an increase in node degree in mice (39) Additionally, connectomics analysis has been used to analyze differences in the LCN structure between sheep and mouse bone, albeit on a limited number of samples (40). The network of sheep bone was more regularly organized but less connected than mouse bone, but properties such as edges per node and edge length were similar between species.

Changes to the LCN have been observed based on the organization of the surrounding matrix, during aging, disease, and in response to environmental factors. More spherical lacunae are likely to be found in woven bone versus the more organized lamellar bone (41). Osteocytes have been seen to elongate perpendicular to the long axis of bones in amphibians, reptiles, and mammals (42). High fat diet caused an increase in lacunar area in mice (39). The LCN also changes with aging, as lacunae become flatter and the canaliculi become more interconnected with maturity, a trend that reverses once bone is aged (43). There are changes to the LCN in osteogenesis imperfecta (OI) as OI mice have been observed to have more spherical lacunae with more canaliculi than wild-type mice (44). Mechanical unloading also results in changes to the LCN. Sciatic neurectomy to immobilize one limb in growing rats resulted in lower lacunar density and volume (45). Similarly, growing mice were found to have a reduced cell volume and number of processes with sciatic neurectomy in both cortical and cancellous bone (46). It is important to note that these experiments were both done in growing rodents. Immobilized female patients had a lower osteocyte density and reduced connectivity than postmenopausal controls (47). Fluid flow as determined by finite element analysis (FEA) was shown to decrease in ovariectomized rats that had lower lacunar density and porosity (33).

The LCN may have a direct effect on bone quality. In cases of spaceflight where the lacunar volume was shown to decrease and become more spherical, nanoindentation indicated that the hardness and stiffness of the matrix also decreased (48). Another study used nanoindentation to assess the area close to (1 to 5 µm) and further away from the lacunae (16 to 20 µm) in ovariectomized rats with treatment. While there were no differences between treatment groups (PTH, alendronate, raloxifene, PTH and alendronate, and PTH and raloxifene), Young’s modulus was lower in the perilacunar region compared to the area further away (49). Modulus was also higher further away from the lacunae and canaliculi in healthy 4-month old female rats (50). Mounting evidence supports that actions coordinated by osteocytes in the LCN directly impact matrix quality.

The pericellular matrix (PCM) surrounds the osteocyte and separates the cell from the walls of the lacunae and canaliculi. This matrix contains proteoglycans and hyaluronic acid and may amplify the impacts of mechanical loading to allow osteocyte to sense more load than what would be calculated by tissue strain alone. Tethering elements between the matrix wall and the cells that could amplify force through shear drag forces in response to fluid flow were first postulated with computational modeling (51) prior to transverse elements between the matrix wall and cell being visually confirmed with TEM imaging (52). Perlecan has been speculated to form the tethering elements in the PCM. MLO-Y4 cells express perlecan protein and immunogold labeling indicated the presence of perlecan along the osteocyte bodies and walls of the canliculi (53). Perlecan deficient mice have shown higher solute diffusivity, but lacked the anabolic response to *in vivo* tibial loading (54) indicating its importance for mechanical sensing (54). Additionally, integrins have been speculated to form part of the PCM and affect the osteocyte response to mechanical stimulation. TEM images have indicated the canalicular walls may have protrusions into the pericellular space (55, 56). A theoretical model incorporating tethering elements along with focal adhesion complexes mathematically predicted a high amplification of strain that was an order of magnitude higher than previous strain amplification models (56). This focal adhesion complex has been speculated to be *β*3-integrin as immunohistochemistry has indicated the presence of *β*3-integrin along the walls of canaliculi of murine cortical bone (55). *In vitro*, inhibition of *α*v*β*3 integrin attachment sites in MLO-Y4 cells reduced the Ca^2+^ response to probe stimulation (57). Structured Illumination Super Resolution Microscopy has found membrane proteins associated with mechanotransduction to be colocalized with *β*3-integrin foci *in vivo*, though this did not find a colocalization with connexin 43 (58). The PCM may also alter with age. Osteocytes isolated from aged mice were able to produce less PCM than osteocytes from young mice *in vitro*. Aged cells also had fewer plasma membrane disruptions than young cells in response to fluid shear stress, indicating the mechanical response may be dependent on the PCM (59). This is an aspect of osteocyte control of the environment that needs to be further studied.

## *In Vitro* Models of Perilacunar Remodeling

Due to their preference of remaining embedded within the bone matrix, osteocytes have proven difficult to study when removed from their natural enclosure. Indeed, studies on primary osteocytes have demonstrated complications including low yield, poor viability when grown in 2D culture, and limited dendrite formation. Therefore, most studies to date have utilized immortalized cellular models of osteocytes. These cell lines represent various stages of the osteocyte life cycle, including late transitioning osteoblasts, early osteocytes, late osteocytes, and lines that gradually differentiate through all three stages. Despite being derived from osteocytes, each cell line responds differently to mechanical, endocrine, and paracrine signals. Therefore, we will begin our analysis of osteocyte matrix modeling and remodeling by examining what has been learned using *in vitro* models.

### Cellular Models of Osteocytes

The most frequently used cell line in osteocyte research is the MLO-Y4 line. These cells were derived from the long bones of female mice and immortalized using an SV40 T-cell antigen coupled to the osteocalcin promoter (60). MLO-Y4 cells are mechanosensitive, as studies utilizing fluid shear stress have demonstrated robust increases in intracellular calcium currents, ATP production, and release of prostaglandin E2 (PGE2) and nitric oxide (NO) (61–63), all of which are essential components of the osteocyte response to mechanical stimulation. Additionally, they express large amounts of connexin 43 (Cx43) and produce a dendritic network. In response to short-term unidirectional and oscillatory fluid flow, MLO-Y4 cells increase RANKL expression while greatly increasing OPG expression, resulting in a decrease in the RANKL/OPG ratio (64, 65). This finding may indicate that osteocytes respond to loading by reducing osteoclast activity through paracrine signaling. Importantly, MLO-Y4 cells do not typically express Sost, a potent inhibitor of bone formation by osteoblasts. This shortcoming is also noted in MLO-A5 cells, a model of late transitioning osteoblasts (66). Interestingly, long-term fluid shear may increase Sost expression in MLO-Y4 cells despite their lack of natural Sost expression, although conflicting evidence exists (67, 68). In terms of anabolic functions, conditioned media taken from MLO-Y4 cultures increases alkaline phosphatase (34) and osteocalcin (OCN) expression in osteoblasts, indicating the presence of additional secreted factors that increase osteoblast activity (69).

Two models of differentiated osteocytes are the Ocy454 and IDG-SW3 cell lines. Each of these lines utilizes an interferon-*γ*-driven T-cell antigen promoter to induce immortalization followed by temperature-driven differentiation. The mechanical response of Ocy454 cells to fluid shear are more variable than MLO-Y4 cells, with fewer cells demonstrating increased calcium currents with occasional high magnitude calcium waves (70). Unlike MLO-Y4 cells, Ocy454 osteocytes express abundant DMP1 and Sost, and Sost expression can be lowered by fluid shear (68). Increasing the duration of fluid shear gradually increases Sost expression and the RANKL/OPG ratio in both lines (68). Like Ocy454 cells, the IDG-SW3 cell line expresses classic osteocytic genes when they reach maturity. In the early stages of differentiation, IDG-SW3 cells express osteoblastic genes including ALP and type I collagen (col1a1) (71). As they transition into early osteocytes, dentin matrix protein 1 (DMP1), matrix extracellular phosphoglycoprotein (MEPE), and phosphate-regulating neutral endopeptidase (Phex) levels increase (71). Finally, as late osteocytes, IDG-SW3 cells begin expressing high levels of Sost and fibroblast growth factor 23 (FGF23), demonstrating their utility in studying osteocyte paracrine and endocrine signaling (71).

While osteoblasts and osteoclasts are the classic cell types involved with forming and shaping bone, emerging research has demonstrated that many of the cues that drive these cells may also trigger osteocytes to participate in these functions. The process of bone matrix alteration by osteocytes is currently known as perilacunar remodeling (PLR), a concept that is gaining popularity in the bone community. Utilizing the idea that osteocytes also modify their activity in response to cues that would normally change bone mass, we next examine how bone-altering signals may modify osteocyte function to alter their surrounding extracellular matrix using the aforementioned *in vitro* models.

### Osteocyte Responses to Endocrine, Paracrine, and Mechanical Stimuli

One of the most important signals the bone receives is from parathyroid hormone (PTH), a peptide hormone secreted from the parathyroid gland in response to low serum calcium. In addition to increasing calcium absorption from the intestine, sustained elevations in PTH are known to cause mineral release from the bone, as seen in hypercalcemia of malignancy and chronic kidney disease (72, 73). Studies using IDG-SW3 cells have demonstrated that PTH upregulates ATPase H+ Transporting V0 Subunit D2 (ATP6V0D2), a proton pump on the cell membrane that acidifies the extracellular environment, indicating that osteocytes can acidify their extracellular environment to degrade mineral (74). PTH-related Peptide (PTHrP) has also been shown to stimulate acidification of the osteocyte extracellular environment by upregulating ATP6V0D2 during lactation, and this process is dependent on intact PTH signaling in osteocytes (75). IDG-SW3 cells naturally upregulate several osteoclastic genes throughout their 28-day differentiation including tartrate-resistant acid phosphatase (TRAP), carbonic anhydrase I and II (CA1/2), and cathepsin K (CTSK), indicating that mature osteocytes are poised to participate in PLR (76). While matrix acidification is required for mineral removal, it also promotes the collagenolytic activity of CTSK, indicating that osteocytes can degrade both mineral and collagen (77, 78). In addition to PTH, Sost signaling has also been shown to upregulate TRAP, CA, and CTSK in neighboring MLO-Y4 osteocytes (79). Therefore, osteocytes may increase the bone resorbing activity of nearby osteocytes in addition to reducing osteoblast activity *via* Sost signaling (76, 79). Finally, TGF*β* also upregulates several osteoclastic genes *via* the YAP/TAZ signaling pathway in MLO-Y4 and Ocy454 cells. In MLO-Y4 cells, treatment with TGF*β* results in extracellular acidification and upregulation of CTSK and matrix metalloproteinase 13 and 14 (MMP13/14) while glucocorticoid treatment decreases MMP13 expression (80). A similar finding was shown in Ocy454 cells, which upregulated CTSK and MMP14, but not MMP13 (78). While these two osteocyte models differ slightly in their responses, they each suggest that osteocytes participate in matrix remodeling by adopting an osteoclast-like phenotype.

As mentioned above, mechanical loading alters osteocyte signaling to osteoblasts and osteoclasts. However, whether loading influences the process of PLR remains unclear. When fluid shear stress is applied to MLO-Y4 cells, increased E11/gp38 expression drives increased dendrite formation and elongation (80). For this process to occur *in vivo*, however, osteocyte dendrites must express genes that allow them to degrade local mineral and collagen to extend through the bone. Indeed, a recent study seeding IDG-SW3 cells into an MMP-sensitive hydrogel demonstrated increased dendricity, Cx43, and MMPs 2 and 13 throughout differentiation (81). These cells also maintained elevated ALP expression through day 28 of differentiation while ALP expression diminishes in 2D culture. Another study in 3D culture demonstrated that MLO-Y4 cells display increased col1a1 expression over time (82). Therefore, 3D culture models may be necessary to capture the ability for osteocytes to form matrix components. However, studies examining perilacunar modeling and remodeling by osteocytes in 3D cell culture with loading or other physiologic stimuli remain to be performed. Altogether, these *in vitro* findings suggest that bone-forming osteoblasts can differentiate into mechanosensitive osteocytes that coordinate the activities of osteoblasts and osteoclasts, and eventually gain osteoclastic resorptive abilities. Strikingly, while osteocytes reduce their osteoblastic activity over time, these functions are not entirely lost as they mature in a 3D environment. Therefore, further research probing the ability for osteocytes to form mineral and collagen are imperative to understand the contribution of osteocytes to the microstructure and overall integrity of bone.

## Modification of Mineral by the Osteocyte

Osteocyte modification of the mineral in the surrounding matrix has been observed in cases where PLR removes mineral such as in lactation (83) and hibernation (84), and lack of PLR can result in hypermineralization such as in the case of exposure to microgravity (48). This has been supported by changes to lacunar area. In the case of lactation, it has been suggested that the osteocyte can also replace the mineral in its surrounding bone if recovery after weaning is allowed, as double fluorochrome labeling has indicated new mineral formation around the osteocyte (75). The osteocyte can alter the overall porosity of bone by either removing or adding mineral to its lacunae. The osteocyte network appears to influence the quality of the mineral as well. Using small angle X-ray scattering (SAXS) combined with confocal microscopy, a study showed that in areas with a high density of osteocytes that were well aligned, the mineral platelet thickness and particle orientation was higher than is less organized areas (85). The mineral thickness and particle orientation were lower in the areas closer to the lacunae themselves, indicating that the osteocytes may control the quality of the mineral over time (85). In another study looking at mice that underwent treadmill running, the mineral to matrix ratio (MMR) of the matrix surrounding the osteocyte was lower than the MMR of the matrix further away, indicating the osteocyte altering its bone matrix (86). Interestingly, mice that underwent treadmill running and showed lower MMR in their perilacunar region had higher post-yield work in bending tests of their tibiae, indicating that PLR may improve bone’s overall mechanical properties (86). An effect on mechanical properties has also been observed elsewhere as the elastic modulus as measured by microindentation of the bone decreased with lactation (87). Thus, changes to the mineral by the osteocyte may affect overall bone quality.

## Collagen Production and Alteration by Osteocytes

Type I collagen is the most prevalent organic component of the bone extracellular matrix and provides the tissue with tensile ductility and fracture toughness by limiting crack formation and propagation (88–90). Collagen is primarily produced by osteoblasts during bone formation alongside mineral. The helical structure of collagen is composed of Gly-X-Y repeats where X and Y are typically proline and hydroxyproline, respectively (91). Collagen consists of two pro-*α*1 and one pro-*α*2 peptide chains that are translated by ribosomes embedded within the endoplasmic reticulum (ER) membrane. Next, post-translational modifications including hydroxylation of proline and lysine residues and glycosylation of some prolines occurs within the ER. The chains twist into a triple helix and are shuttled to the Golgi apparatus as procollagen. Upon secretion from the osteoblast, the N- and C-terminal domains are cleaved, forming tropocollagen. Finally, tropocollagen strands self-assemble into fibrils and neighboring tropocollagen molecules are crosslinked at their hydroxylysine residues by lysyl oxidase, stabilizing the fibrillar structure (92).

The overall quality of collagen is dependent on the correct level of post-translational modifications, proper crosslinking, incorporation into the bone, and alignment within the bone tissue. Importantly, the alignment of collagen fibrils is related to the types of loads that each bone experiences. During physiologic loading of the lower limb, the anterior portion of the femur and tibia typically experiences tension while the posterior portion is under compression (93). Studies utilizing polarized light microscopy have determined that collagen fibrils tend to be aligned perpendicular to transverse sections of bones under tension while they are aligned parallel to transverse sections in compressive regions. Intriguingly, collagen fibrils tend to align with the major axis of osteocytes and their lacunae (41). Additionally, it has been shown that osteoblasts initially secrete disorganized collagen that eventually aligns with the osteoblast major axis or the axis under the greatest mechanical strain (94, 95).

While osteoblasts follow mechanical cues from their environment to determine collagen orientation and placement, mechanosensory cues from osteocytes may also be required to instruct osteoblast collagen deposition. Further, it stands that osteocytes themselves may be responsible for forming and aligning collagen in the perilacunar region. One of the earliest studies examining this possibility placed bones from egg-laying hens in media containing radiolabeled proline, a highly prevalent amino acid in all collagens (96). In hens fed a calcium-rich diet after egg laying, it was reported that osteoblasts and osteocytes demonstrated widespread uptake of proline, indicating that osteocytes may replenish matrix collagen following lactation (97). Modern intravital imaging studies have also demonstrated that early osteocytes may be able to synthesize parts of the collagen matrix surrounding their lacunae while also exerting mechanical forces on the existing collagen matrix (8). Eventually, this process results in a collagenous matrix that aligns with the major axis of osteocyte lacunae, but whether this process is mechanically driven remains unknown.

As discussed earlier, *in vitro* models of osteocytes have the capacity to degrade collagen in response to catabolic stimuli including PTH. The importance of this finding has also been established *in vivo*, as lactating mice fail to resorb mineral from their lacunae if collagen degrading genes including CTSK and MMP-13 are knocked out in osteocytes (98, 99). Therefore, collagen degradation is an essential step in perilacunar remodeling. Additionally, MMP-13 expression by osteocytes is critical to maintenance of bone fracture toughness, or the ability of bone to resist crack formation and propagation, a property that is highly dependent on proper collagen incorporation and crosslinking (99, 100). There are implications that TGF-*β* may be involved as well. It has been demonstrated that inhibiting TGF-*β* receptor pharmacologically in mice resulted in a reduction of gene expression of genes associated with PLR and reduced canaliculi length (76). The same study examined a knock-out mouse of osteocyte specific TGF-*β* receptor in bone which resulted in a similar decline in PLR gene expression and decrease in canalicular length and lacunar–canalicular area. Fracture resistance was notably lower in the knock-out mice (76). Taken together, there is striking preliminary evidence to warrant a deeper investigation of the osteocytes ability to modify, align, and produce collagen within their lacunae. Future work may require the use of 3D scaffolds in order to capture these effects *in vitro* while *in vivo* studies will likely benefit from the use of emerging techniques to analyze bone composition and mechanical properties on the microscale in animal models of post-lactation recovery, space flight, and other instances of mineral challenge. Furthermore, while collagen is the most prevalent protein in bone, genetic knockouts of non-collagenous proteins (NCPs) including biglycan, fibrillin-2, and bone sialoprotein among others have demonstrated altered microarchitecture and/or reduced mechanical properties (101–103). Biglycan in particular is required for proper collagen assembly into organized fibrils, and knockouts resemble the phenotype of Ehlers–Danlos syndrome (104). However, material tests such as fracture toughness testing and tissue-level analyses have largely not been performed on these genetic models. While NCPs are known to impact bone formation, whether osteocytes can interact with and alter NCPs, or whether NCPs control the ability for osteocytes to model and remodel their surrounding matrix remains largely unknown. Taken together, understanding the full extent of the osteocytes capabilities will require a combination of robust cellular models, modern imaging modalities, and tissue-level analyses that can distinguish material, structural, and compositional properties on the micro- and nano-scales in and around the LCN, enhancing our ability to devise new treatments for bone diseases.

## Conclusion

**Figure 1:**
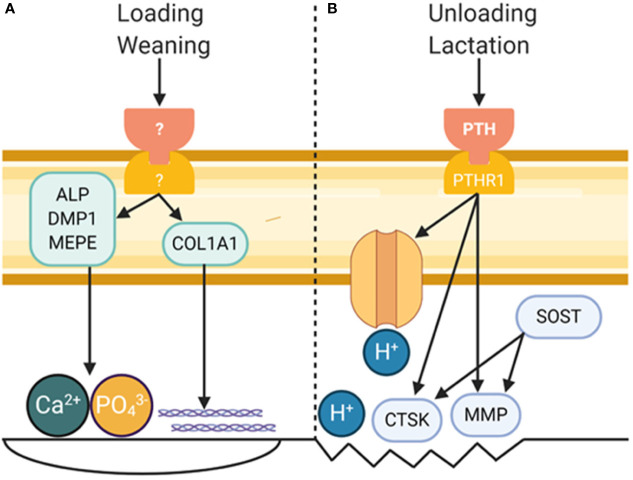
Role of Osteocytes in Modifying Bone Matrix Properties. **(A)** Mechanical loading and wearing are known to increased bone formation, and osteocytes are known to play a critical role in each. While a definite pathway leading to bone formation by osteocytes remains to be discovered, a small amount of bone formation by osteocytes could have a profound effect on bone material properties. **(B)** Perilacunar remodeling by osteocytes occurs in response to unloading and lactation, processes in which PTH is a major player. This results in decreased pH in the perilacunar space and increased expression of collagenolytic enzymes. Figure created with BioRender.com.

The osteocyte has a profound effect on the bone matrix through signaling to osteoblasts and osteoclasts and by directly modifying its environment (**Figure 1**). The structure of the LCN relates to the structure and quality of the surrounding matrix. There is also *in vitro* and *in vivo* evidence indicating the osteocyte can directly modify mineral and collagen in its surroundings. Thus, the osteocyte must be considered when examining the effects of disease and treatments on the bone matrix.

## Author Contributions

AC, JD, and JW planned and wrote the document. All authors contributed to the article and approved the submitted version.

## Funding

This work was supported by the National Institutes of Health [JW: AR072609; AC: AR065971; JD: DK121399].

## Conflict of Interest

The authors declare that the research was conducted in the absence of any commercial or financial relationships that could be construed as a potential conflict of interest.

The reviewer CW declared a shared affiliation with one of the authors, JD, to the handling editor at time of review.
